# The Emerging Field of Psychedelic Psychotherapy

**DOI:** 10.1007/s11920-022-01363-y

**Published:** 2022-09-21

**Authors:** Gregory S. Barber, Scott T. Aaronson

**Affiliations:** 1 Department of Psychiatry, University of Maryland Medical Center, Baltimore, MD USA; 2 Institute for Advanced Diagnostics and Therapeutics, Sheppard Pratt Health System, 6501 N. Charles Street, Towson, MD USA

**Keywords:** Psychedelics, Psychedelic-assisted therapy, Psilocybin, MDMA, Treatment resistant depression

## Abstract

**Purpose of Review:**

Few treatments are available for patients with mood disorders or post-traumatic stress disorder (PTSD) who have already failed multiple interventions. After several decades when research into psychedelics was effectively halted by federal legislation, the past several years have shown the re-emergence of thoughtful investigations studying the utility of compounds such as 3,4-methylenedioxymethamphetamine (MDMA) and psilocybin.

**Recent Findings:**

Several studies have coupled the safe administration of psychedelic compounds in a controlled environment after several hours of preparation of study participants and followed by multiple sessions to integrate the psychedelic experience. The improvement participants experience appear related to the often profound perspective changes experienced and seem unlike the improvements seen in the currently available care paradigms. Studies cited include treatment resistant depression, end of life despair, and PTSD.

**Summary:**

Psychedelic psychotherapy, a unique remarriage of biological therapy and psychotherapy, has the potential to transform mental health care.

## Introduction

Psychedelic-assisted psychotherapy has garnered large amounts of attention in recent years as the next potential breakthrough treatment for psychiatric illnesses. Despite unprecedented levels of psychopathology worldwide, the field of psychiatry has produced relatively few novel interventions in recent decades. Globally, depression is a top five cause of disability, affecting about 5% of the world’s population [1], while PTSD has a global prevalence of 3.9%, with high-income countries having a higher burden of PTSD compared to low-income countries [2]. Psychedelic-assisted psychotherapy offers the hope of a new treatment whose rapid onset and enduring efficacy could outpace that of other psychiatric treatments. This promise, combined with psychedelics’ cultural and historical significance, has led psychedelic therapies to garner huge amounts of attention for a treatment that remains in an investigational stage. Psychedelic therapies are discussed on the front page of the New York Times [3, 4], best-selling popular books [5], documentaries [6], and beyond. Headlines declare a “new age for psychiatry” while political leaders from across the political spectrum have endorsed therapeutic access to these once maligned and still criminalized compounds [7, 8].

While psychedelics have been used in traditional religious and cultural practices for millennia, interest in their potential therapeutic applications traces its origins to the mid-twentieth century. At this time, countercultural forces combined with the rise of psychopharmacology to fuel optimism about psychedelic therapies. Between the 1950s and 1970s, psychedelics like lysergic acid diethylamide (LSD) and psilocybin were given to tens of thousands of patients to treat conditions like alcohol abuse, depression, anxiety, and end-of-life distress [9]. Research into these substances largely halted once the Nixon administration placed them on schedule 1 of the Controlled Substances Act of 1970, where they remain to this day. Between the mid-1970s and mid-1980s, some therapists became interested in the promise of 3,4-methylenedioxymethamphetamine (MDMA) to kickstart psychotherapy [10]. But like psilocybin and LSD before it, MDMA’s therapeutic use was largely shut down when it too was made a schedule 1 substance in 1985.

## Types of Psychedelic Substances

Psychedelics are a broad class of substances defined by their ability to induce altered states of consciousness, including changes in perception, thinking, and feelings. While hundreds of naturally-occurring and lab-made psychedelic compounds exist, only a handful have been studied therapeutically in clinical trials. Though psilocybin and MDMA have received the most attention for their potential therapeutic applications in recent years, other common psychedelics, including LSD, dimethyltryptamine (DMT), ayahuasca, ibogaine, and mescaline have also been studied to a lesser extent. The commonly used dissociative anesthetic ketamine, which has psychedelic properties, has also found a role in many psychiatric settings. Here, we briefly describe the differences between two subclasses of psychedelics, the empathogens and the classical psychedelics.

### MDMA and Empathogens

MDMA is a synthetic amphetamine derivative with action on multiple neurotransmitter systems, including norepinephrine, serotonin, dopamine, and oxytocin. MDMA is in the subclass of psychedelics called “empathogens,” named for their ability to promote attachment, trust, empathy, and interpersonal connectedness [11]. MDMA can also produce a sense of meaning and feelings of euphoria. MDMA may cause subtle perceptual changes that are generally less intense than those caused by the classical psychedelics. It also affects emotional perception, making users slower to perceive anger in other people and producing stronger responses to positive emotions [12].

MDMA has a variety of physiological effects. Stimulant-induced hypertension and tachycardia may lead to adverse cardiac outcomes [13]. Hyperthermia may lead to heat injuries [14], while hyponatremia can cause to seizures [15]. Neurotoxicity [16] and hepatotoxicity [17] are also possible. Fortunately, in clinical trials, MDMA has not been associated with severe toxicity, nor have research participants displayed abuse or drug-seeking behaviors after taking MDMA. While MDMA is generally considered safe relative to other recreational drugs [18], severe reactions are possible. Over the course of 2 decades in 4 countries, one study found 1400 drug deaths involving MDMA [19]. Of these, 13–25% were attributable to pure MDMA alone. It is important, however, to recognize that the risks associated with recreational MDMA use — in which drug purity and identity, as well as the potential presence of other adulterants, cannot be guaranteed — are different from the risks of MDMA in clinical settings.

### Psilocybin and Other Classical Psychedelics

The classical psychedelics, which include psilocybin, LSD, mescaline, and dimethyltryptamine (DMT), are defined by their agonism of the 5HT-2A serotonin receptor. While the classical psychedelics each have subtle distinctions in their effects, they tend to induce changes in perception (e.g., illusions, distortions, amplifications, or hallucinations in multiple sensory modalities), increased cognitive flexibility, and intense emotions [11]. In certain patients who take sufficient doses, the classical psychedelics can induce mystical experiences, ego dissolution, and a sense of the interconnectedness of all beings. They also have the capacity to provoke intense anxiety and dysphoria.

In terms of safety, classical psychedelics including psilocybin are considered relatively well-tolerated and low risk. In one of the larger clinical trials investigating psilocybin’s use in major depressive disorder, there were no serious adverse medical events [20]. Only one patient experienced a transient elevation in blood pressure, while 33% of patients experienced self-limited headaches. There were no serious cardiac or neurological events. Other risks include nausea and vomiting shortly after ingesting psilocybin. While experts consider a personal or first-degree family history of psychosis to be a contraindication for psilocybin-assisted psychotherapy, clinical trials thus far have not been associated with episodes of psychosis among research participants. There have also been no reports of completed suicides among clinical trial participants thus far. Hallucinogen persisting perception disorder, in which psychedelic users re-experience perceptual distortions and dissociative states for months or years after taking a psychedelic, is an under-studied risk whose prevalence and impact on patients is poorly understood [21].

Beyond clinical trials, much of the knowledge about classical psychedelics’ safety comes from epidemiological studies among recreational users. In these settings, psychedelics account for the least emergency room usage of all recreational drugs [22]. Non-clinical use of classical psychedelics, moreover, has been associated with reduced suicidality and psychological distress among the general population [23]. The risk of abuse of psilocybin is considered low and there is no physical dependence [24].

## Modern Psychedelic Research

### MDMA

While interest in psychedelics between the 1970s and 1990s never entirely disappeared, the new millennium brought about a renaissance in psychedelic research. Between 2004 and 2017, there were six positive phase 2 clinical trials investigating the use of MDMA for PTSD [25•]. Averaging across these trials, MDMA had a large effect size compared with control groups (Cohen’s *d* = 0.8) on PTSD symptoms, with a mean difference in CAPS-IV scores of − 22.0 points. Depending on the specific trial design, patients received MDMA or control in two or three dosing sessions, which were separated by a month. All the trials involved several psychotherapy sessions before the dose, and three integration sessions after each dose.

On the basis of these results, MDMA gained the FDA’s Breakthrough Therapy Designation in 2017. During this time, one important phase 2 clinical trial demonstrated the feasibility of maintaining consistent protocols across multiple treatment sites [26•] in preparation for a phase 3 trial. The first phase 3 clinical trial, published in 2021, compared active MDMA-assisted psychotherapy in 46 patients with placebo + therapy in 44 patients [27••]. This trial demonstrated a large effect size of MDMA-assisted therapy compared to placebo with therapy (Cohen’s *d* = 0.9) and had a similar structure for pre- and post-dose therapeutic sessions as the phase 2 trials described above. Of note, patients with the dissociative subtype of PTSD, which is often considered more difficult to treat, improved at least as much as patients with the non-dissociative subtype. Moreover, patients in the placebo group also showed significant improvements in PTSD, supporting the efficacy of psychotherapy (as well as the efficacy of set and setting independent of drug effects) in treating PTSD.

### Psilocybin

Meanwhile, 2016 brought a pair of double-blind, randomized controlled clinical trials with a total of 80 patients that looked at psilocybin-assisted psychotherapy’s effects on anxiety and depression in patients with life-threatening cancer [28•, 29•]. These studies found strong reductions in both depression and anxiety symptoms, as well as improvements in quality of life and spiritual well-being, from psilocybin-assisted psychotherapy. That same year, another pilot trial found success with psilocybin for treatment-resistant depression [30•]. Moreover, the studies helped establish the safety of psilocybin, which was well-tolerated and did not cause any serious negative physiological or psychological consequences. Other research has supported psilocybin’s capacity to treat tobacco use disorder [31], alcohol use disorder [32], and obsessive compulsive disorder [33].

**Fig. 1:**
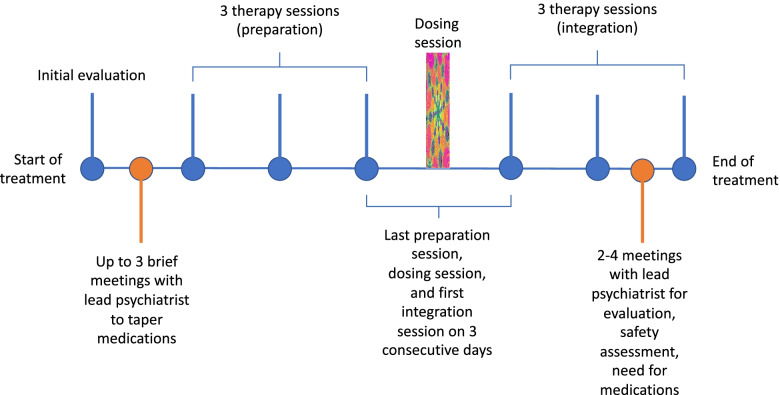
Psychedelic-assisted psychotherapy consists of 3 stages: preparation, dosing, and integration. Though there is some variation across clinical trials, in general, participants undergo an initial evaluation followed by meetings with the psychiatrist to taper off their medications. In preparation, the patient meets with the lead therapist 3 times over the course of 3 weeks, with the co-therapist joining for the final preparation session. Preparation focuses on getting the patient comfortable with the potential range of experiences they may experience under the influence of psychedelics, exploring expectations and setting intentions, and developing strategies for navigating any challenges that might emerge during dosing. Integration also has sessions with the therapist over 3 weeks, with the co-therapist joining only for the first integration session. In integration, the goal is to consolidate the insights gleaned during dosing and help the patient apply these changes to their daily life. Throughout this process, the therapist practices a non-directive, present-focused technique which encourages the patient to engage with all aspects of their current experience

In one of the largest clinical trials so far on psilocybin-assisted psychotherapy for major depressive disorder (*n* = 27), 71% of participants had a clinically significant response to psilocybin 4 weeks after a single dose, while 54% of participants achieved remission from depression over the same time, corresponding with large effect sizes (*d* = 2.3) [20••]. Of note, this was a crossover trial, which can inflate response rates. Another recent trial found that psilocybin was non-inferior to the commonly prescribed anti-depressant escitalopram, with 30 patients receiving psilocybin and 29 patients receiving escitalopram [34••]. Across these trials, positive benefits often endured for months after just a single psilocybin dose. Many patients have commented that the psilocybin session was among most meaningful experiences of their life [35]. Table 1 provides some details of the clinical trials described above. While some variation exists between psychedelic protocols, the therapeutic process tends to follow a general timeline and structure, which is detailed in Fig. 1.

**Table 1:** Details of the clinical trials on important psychedelic clinical trials.

Study authors and year	Number of participants	Substance studied	Indication	Study design	Control group	Outcome
Mithoefer et al. [25•]	105	MDMA	PTSD	Pooled analysis of 6 phase 2 clinical trials; blinded crossover design with 2 dosing sessions and the potential for a supplemental dose during the dosing session; also potential for an open label extension after the initial 2 active doses	Either placebo or low dose MDMA	Large effect (*d* = 0.8) on CAPS-IV total score reduction in the treatment group compared to control. After 1–2 months, 54.2% in the active treatment group no longer met PTSD criteria vs 26% in the control group
Mithcell et al. 2021	90	MDMA	PTSD	Phase 3, double-blind, placebo controlled clinical trial	Manualized therapy with placebo	Large effect (*d* = 0.91) on CAPS-5 total score reduction in the treatment group compared to control. Significantly reduced clinician-rated functional impairment (d = 0.43). Significant improvement in patients with both dissociative and non-dissociative subtypes of PTSD
Griffiths et al. [28•]	51	Psilocybin	End-of-life anxiety and depression associated with life-threatening cancer	Randomized, controlled, double blind crossover trial	Low-dose psilocybin	Large and sustained reductions in depressive and anxiety symptoms, with a mean effect size of *d* = 0.82 across 11 measures in the high-dose first group compared to the low-dose first group. Between both groups, at 6 months 65% achieved remission for depression and 57% achieved remission for anxiety
Ross et al. [29•]	29	Psilocybin	End-ofend of lifelife anxiety and depression associated with life-threatening cancer	Randomized, controlled, double blind crossover trial	Niacin	Significant differences in anxiety and depression compared to baseline at all follow up timepoints up to 26 week follow-up 83% of participants in the psilocybin-first group achieved antidepressant response at 7 weeks after the dose
Carhart-Harris et al. [30•]	12	Psilocybin	Moderate to severe MDD	Open-label feasibility trial	None	Marked reduction in depressive and anxiety symptoms relative to baseline at 1 week and 3 months as measured by the QIDS
Davis et al. 2020	27	Psilocybin	MDD	Randomized, wait list controlled phase 2 clinical trial	Wait list (8 week delay to start of treatment compared with immediate treatment)	Large effect sizes at weeks 5 and 8 (d 2.5 and 2.6, respectively) in the immediate group compared with the delayed group as measured by reductions in the HAM-D. Overall, 54% of patients were in remission at 4 weeks after active treatment
Carhart-Harris et al. [34•]	59	Psilocybin	Moderate to severe MDD	Double-blind, randomized, head-to-head phase 2 clinical trial	Escitalopram	70% antidepressant response in the psilocybin group and 48% antidepressant response in the escitalopram group based on QIDS scores No significant difference in antidepressant efficacy based on QIDS scores at week 6 between the psilocybin group and escitalopram group

## Psychological Effects of Psychedelics

Patients taking psilocybin and MDMA can have a vast range of phenomenological experiences. In clinical trials, patients are encouraged to look inwards and engage fully with their minds. Preparation sessions emphasize adopting the attitude of “trust, let go, and be open.” The patient will ideally be willing to embrace any content, however challenging or painful, that emerges during the dosing session. The psychedelic experience is often considered ineffable and can take the form of intense memories, surrealist imagery, contact with entities, profound insights, or any number of other possibilities. Ultimately, there is no predicting how one’s psychedelic experience will unfold. In preparation for the psychedelic experience, patients will ideally learn to not fight the process.

While more research is required to determine the “active ingredient” of classical psychedelic therapies, many consider such intense subjective effects, including mystical experiences, to be essential for therapeutic benefit [36]. However, others disagree, and attempts are underway to engineer “non-hallucinatory” psychedelics which achieve therapeutic benefit while avoiding subjective effects [37]. In our experience, patients benefit most from psychedelic treatments when they’ve had the type of phenomenological experience that allows them to reframe and reorient to the strongly held beliefs which underly their mental illness, thus supporting the view that the subjective effects do substantially (though perhaps not entirely) mediate therapeutic benefits.

One way of thinking about the psychological mechanisms of psychedelics’ action is that they function as “belief relaxers” [38•]. Underlying various forms of psychopathology are strongly held, maladaptive beliefs which guide the ways patients perceive the world, other people, and themselves. Psychedelics act to “relax” these strongly held beliefs. With rigorous psychotherapeutic integration, patients begin to consider new perspectives and are able to restructure their habitual ways of approaching the world. The intensity of psychedelic states, including the mystical experiences they may occasion, allows old beliefs to “weaken,” making space for new beliefs to enter the mind in a deep and meaningful way. From here, patients are able to integrate a new sense of themselves in relation to life’s challenges.

## The Future of Psychedelic Treatments

Psychedelics remain in an early stage of research. In the era of modern psychedelic research, participants numbering only in the hundreds have received MDMA and psilocybin in clinical research settings. While the early clinical trials have been impressive and demonstrate the promise of psychedelic treatments, findings have yet to replicate on a large scale. The sole phase 3 clinical trial on MDMA for PTSD so far (Mitchell et al., 2020) itself included just 90 patients. Fortunately, further phase 3 clinical trials for MDMA are underway, and phase 3 trials on psilocybin for PTSD are also currently being planned. For psilocybin, the largest trial thus far has been a phase 2, dose finding study where 233 patients were randomized to 1mg, 10mg, or 25mg of psilocybin. [39]. A phase 3 clinical trial on psilocybin for treatment-resistant depression with likely start by the end of 2022.

One major area for future research is the generalizability of these clinical trials. The large majority of participants in these trials are white individuals being treated in carefully controlled research settings [40]. Whether people from diverse socioeconomic, racial, and ethnic backgrounds respond to and benefit from psychedelic therapies in the same way as the participants in clinical trials so far remains a crucial open question. Moreover, the comparative risks and benefits of MDMA and psilocybin versus other novel psychiatric treatments, such as ketamine and various neuromodulation techniques, remain unknown. However, there is promise that psychedelics could outperform existing treatments. Psilocybin, for example, has demonstrated rapid and sustained antidepressant effects without tolerance or dependence in trials so far. Compared with ketamine, whose effects wear off after a few weeks of treatment, often requiring repeated dosing and carrying the risk of dependence and tolerance, psilocybin may carry a more favorable risk–benefit profile, though more research is required to definitively determine this. For MDMA, one pontential major comparative advantage is its efficacy in dissociative PTSD, an area for which psychiatry has relatively few effective pharmacological treatments. Whether or not these effects will translate into a treatment of dissociation more generally remains unknown.

Aside from ketamine, no psychedelic treatments have yet been approved for therapeutic use by the US Food and Drug Administration. Both MDMA and psilocybin remain on Schedule 1 of the US Controlled Substances Act, which means that officially, they are still considered to lack legitimate medical uses. Should MDMA and psilocybin become FDA approved, they would be reclassified into a less restrictive scheduling category. Researchers at Johns Hopkins have determined that, on the basis of psilocybin’s pharmacological characteristics, including its low abuse liability, it would be most appropriate for a Schedule 4 designation, which would place it in the same category as commonly used benzodiazepines [24]. Similar conversations about the appropriate scheduling level for MDMA are unfolding as research progresses.

Meanwhile, researchers are increasingly exploring the role of psychedelics in other psychiatric conditions. Ongoing or planned trials for psilocybin are exploring its ability to treat bipolar depression, suicidal ideation, depression associated with early Alzheimer’s disease, anorexia nervosa, and mood disorders in early-stage cancer patients (as opposed to late-stage/life threatening cancers). There has also been discussion in the research community on the prospect of using psilocybin to treat personality disorders, including borderline personality disorder [41]. Research with MDMA has for the most part remained focused on its use in PTSD.

At this point, only some research has been devoted to determining patient-specific factors that predict psychedelic outcomes [42, 43]. Our experience in clinical trials suggests it is important to carefully screen patients for co-morbid personality disorders when they are pursuing psychedelic treatments for primary affective disorders. Given the intensity of the psychedelic experience, patients with a poorly integrated sense of self, and those with difficulties in emotional regulation and distress tolerance, may find the psychedelic experience destabilizing. We also suspect that patients who are high in avoidance — independent of the presence of personality disorders — may also have more challenges tolerating the psychedelic state. As research progresses, clinicians will gain a better sense for which patients are most likely to benefit from psychedelic treatments.

Clinicians will also increasingly have to contend with what it means to “get better” after using psychedelic therapies. Some have argued that psychedelics may not actually improve core depressive symptomatology and only help with things like insight and self-awareness [44]. While we agree that some of the benefits of psychedelic treatments may be beyond core symptomatology, this phenomenon does not devalue the treatment. Improvements in insight, relationships, sense of self, and feeling of connectedness can represent major changes for patients suffering from mental illness. Perhaps improvements in core symptoms lag behind such psychological changes as ongoing psychotherapy progressively mobilizes the insights gleaned during the psychedelic experience. Whatever the exact relation, we suspect that the immediate psychological impacts of psychedelics mediate changes in core symptomatology, like the neurovegetative symptoms of depression, in important ways. Future research will hopefully reveal these relationships, but to dismiss psychedelic therapies now, particularly given the positive results of clinical trials — which *are* largely about core symptoms — would be an overly-hasty assessment.

The question of scalability and access also looms over psychedelic therapy’s future [45]. Most psychedelic clinical trials require a team of one supervising psychiatrist plus two graduate-level mental health professionals. A single patient undergoes several therapeutic preparation sessions in advance of the psychedelic session, an 8-h dosing session, and several therapeutic integration sessions following the dosing. Psychedelic therapies are time and labor intensive — particularly the requirement that two therapists devote a full work day to a single patient on the day of dosing. While cost effectiveness, affordability, and accessibility are less problematic in clinical trials, these issues will be major challenges if psychedelic therapies 1 day become available to the general population.

Creative solutions like group psychedelic psychotherapy, which reduces the ratio of clinical staff to patients and thus lowers cost and improves access, are currently being explored [46]. Other developments, including efforts to have insurance companies cover psychedelic treatments, are important steps towards widespread access. Future research should also consider whether the higher costs associated with psychedelic-assisted therapies “pay for themselves” due to their enduring positive benefits and the clinical stability and reduced hospitalization that this may entail. Compared to surgical procedures, which also involve high clinician to patient ratios and involve huge amounts of resources (likely far beyond what is involved here), the economics of psychedelic therapies should not be insurmountable.

Other stakeholders are pursuing non-medical models for psychedelic psychotherapies. Enthusiasm for psychedelic treatments has spurred several movements promoting their decriminalization and legalization. The most notable of these is unfolding in Oregon, where in November 2020 voters approved a ballot initiative legalizing psilocybin and establishing a statewide system of psilocybin therapy clinics. Motivated by the high burden of mental illness and the urgent need for accessible treatments, Oregon decided to allow psychedelics to be administered by facilitators without mental health training, and for people to use psilocybin without a prescription. Generalizing from the success of clinical trials, Oregon has decided to massively expand access to psilocybin. They are apparently doing so without consideration for the role of thoughtfully designed protocols and highly trained mental health professionals in creating the success of those very clinical trials. We firmly believe that the future of psychedelic psychotherapies will involve a collaboration between patients, psychiatrists, and other mental health professionals. We also firmly believe that individualized risk–benefit assessments, performed by skilled clinicians, will be essential to maintaining the quality and integrity of psychedelic therapies in the future.

## Conclusion

Psychedelic therapies represent an exciting opportunity for psychiatry — allowing patients to achieve meaningful improvements in function, symptomatology, and overall outlook in a relatively short amount of time. Though many challenges, several of which we discussed here, lie ahead, we are optimistic about psychedelics’ — and psychiatry’s — future.
